# Understanding the contextual determinants, barriers and facilitators of mental health for adolescent girls and young women in Burundi: Insights from lived experiences

**DOI:** 10.1017/gmh.2026.10233

**Published:** 2026-05-28

**Authors:** Gina Chowa, Joan Wanyama, Rainier Masa, Sumudu Wijesuriya, Annagrace Saufley

**Affiliations:** 1 School of Social Work, The University of North Carolina at Chapel Hill, USA; 2 Global Social Development Innovations Center, The University of North Carolina at Chapel Hill, USA; 3 Centre for Social Development in Africa, University of Johannesburg, South Africa

**Keywords:** Adolescent mental health, culturally adaptive interventions, school-based interventions, gender-responsive programs

## Abstract

Adolescent girls and young women (AGYW) in Burundi face substantial mental health risks stemming from trauma, displacement, bereavement, and sexual violence, compounded by poverty, social upheaval, and limited mental health services. Stigma and marginalization further constrain help-seeking. This study examines the distinct mental health challenges faced by AGYW in Burundi, exploring how socio-cultural and structural factors shape their psychological well-being and identifying key barriers and facilitators to accessing mental health support. Seven focus group discussions (FGDs; total n = 55; 7–8 participants each, ages 15–24) were conducted using purposive sampling to ensure diversity by ethnicity, disability status, and school enrollment. Data was analyzed thematically using a preliminary coding framework. Four thematic domains emerged: (1) contextual conditions shaping mental health; (2) barriers to service access; (3) facilitators for improving access; and (4) intervention needs. Stigma and low community awareness contributed to isolation and silence around distress. Structural barriers, including poverty, food insecurity, and unstable home environments, limit service uptake and educational engagement, with daily survival needs often superseding care-seeking. Facilitators included culturally relevant, community-based approaches and school settings as potential safe spaces for support. Priorities include stigma reduction, community awareness, and school-based delivery models that address gender-based violence and societal norms.

## Graphical abstract

**Figure:**
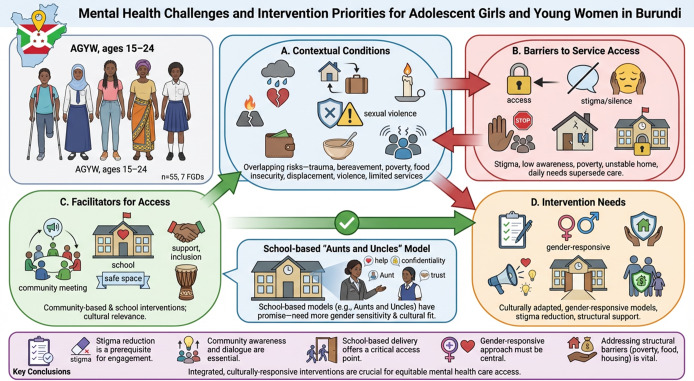

## Impact statement

This study makes a significant contribution to the evidence base on adolescent mental health in low-resource and conflict-affected settings by centering the lived experiences of adolescent girls and young women (AGYW) in Burundi, an understudied population in global mental health research. This study moves beyond prevalence estimates to illuminate the contextual determinants shaping mental health, including structural inequalities, gender norms, economic precarity and exposure to violence by applying phenomenological qualitative methods. Importantly, it identifies both barriers and locally grounded facilitators to mental well-being, offering nuanced insights that are directly actionable for policy and programming. The findings have clear implications for the design of culturally responsive, gender-sensitive and contextually relevant mental health interventions, particularly those integrated within existing community and social protection systems. By amplifying AGYW voices and highlighting pathways for resilience and support, this work advances equitable mental health discourse and provides a critical foundation for targeted interventions and future research in similar.

## Background

Adolescent girls and young women (AGYW) navigate a critical developmental period marked by profound physical, emotional and social transitions. This life stage is associated with heightened vulnerability to mental health challenges, shaped by a complex interaction of hormonal fluctuations, psychosocial stressors and lived experiences (Patel et al., 2018; WHO, 2021). Structural inequities such as gender-based discrimination, socioeconomic disparities and limited access to culturally responsive mental health services further compound these challenges (United Nations Children’s Fund, 2021). Mental health during adolescence and young adulthood is integral to overall well-being, as it influences cognitive, emotional and social development during a formative life phase (WHO, 2021). Common manifestations of mental health concerns among young women include anxiety, depression, low self-esteem and stress-related conditions (Shah et al., 2024). These conditions are driven by both biological influences, including hormonal changes and socio-environmental factors such as peer pressure, cultural expectations and the pervasive impact of social media (Twenge and Campbell, 2018). When unaddressed, these issues can have enduring consequences, affecting educational attainment, interpersonal relationships and long-term life trajectories (Kieling et al., 2011).

### Mental health in Burundi

Burundi, one of the world’s poorest countries in terms of per capita income, has endured a protracted history of armed conflict that has left deep psychological scars across its population (Familiar et al., 2013). The prolonged violence and political instability have contributed to widespread mental health concerns, with individuals frequently reporting symptoms of fear, anxiety, despair, grief and chronic uncertainty as enduring consequences of the conflict (Hassan et al., 2016). Among those most affected are AGYW, who face disproportionate mental health risks due to their exposure to traumatic experiences such as witnessing violence, forced displacement and the loss of family members during the civil unrest (Hall et al., 2014; Tol et al., 2014; Charak et al., 2017). These experiences profoundly impact their emotional and psychological development, contributing to an increased prevalence of depression, anxiety and posttraumatic stress symptoms.

WHO (2021) identifies mental health disorders as a leading cause of disability among young people in Burundi, yet access to care remains limited. Structural barriers, most notably the lack of mental health infrastructure and trained personnel, are compounded by pervasive stigma, which is deeply embedded in cultural norms that marginalize discussions of mental illness, particularly among women and girls. This stigma discourages help-seeking behaviors and fosters a culture of silence and shame. As a result, many AGYW endure mental health struggles in isolation, fearful of judgment, social exclusion, or being perceived as emotionally weak (Hassan et al., 2016).

Beyond the pervasive stigma, the limited availability of mental health services in Burundi significantly exacerbates the mental health crisis, particularly among AGYW. The country faces a severe shortage of trained mental health professionals, with existing resources heavily concentrated in urban centers (Shah et al., 2024). This urban–rural disparity creates substantial barriers for the majority of the population residing in rural areas, where logistical, financial and infrastructural constraints hinder access to even basic mental health care.

As a result, large segments of the population remain underserved, reinforcing systemic inequities in mental health service delivery. When services are accessible, they are typically reserved for individuals presenting with acute psychological conditions such as psychosis or trauma-related disorders. Adolescents and young women with more common, yet less visible, conditions such as anxiety, depression and stress-related disorders are often overlooked (WHO, 2021).

This selective prioritization perpetuates service gaps and contributes to the chronic neglect of everyday mental health concerns. Compounding these challenges are gender-specific barriers that disproportionately affect young women’s ability to seek and receive care. Cultural expectations often position women as primary caregivers, limiting their time and autonomy to prioritize their own well-being. Additionally, restrictive gender norms constrain independent decision-making, further obstructing access to mental health services (United Nations Children’s Fund, 2021). These intersecting structural and gendered barriers underscore the urgent need for equitable, accessible and gender-responsive mental health strategies in Burundi.

### Prevalence of mental health challenges for AGYW in Burundi

WHO (2004) defines mental health as a state of psychological and emotional well-being in which individuals recognize their abilities, manage everyday stressors effectively, engage in meaningful work and contribute productively to their communities. Complementing this definition, the United States Agency for International Development (2024) expands the concept to encompass emotional, cognitive, psychological and social dimensions of well-being. Assessing and understanding the mental health of AGYW in Burundi is particularly critical, given their heightened exposure to a range of intersecting stressors, including poverty, limited access to education, gender-based violence and ongoing geopolitical instability. These factors interact in compounding ways, amplifying psychological distress and undermining emotional resilience. Poverty not only restricts access to basic necessities but also increases exposure to unsafe environments and social exclusion, conditions strongly linked to elevated levels of anxiety and depression (Lund et al., 2010). Educational exclusion further limits AGYW’s potential for personal development and economic independence, contributing to diminished self-worth and pervasive hopelessness (Patel et al., 2018).

Gender-based violence represents an additional and deeply entrenched threat, with experiences of physical, emotional, or sexual abuse associated with long-term mental health consequences, including depression and suicidality among women (Devries et al., 2013). In the context of Burundi, these challenges are further intensified by recurrent political and economic instability, which continues to disrupt social cohesion and community-based support networks. As a result, AGYW remain particularly vulnerable to cumulative trauma and chronic mental health disorders in an environment where access to mental health care is both limited and stigmatized (Hassan et al., 2016).

Mental health conditions among adolescents in sub-Saharan Africa are increasingly recognized as a critical public health concern. Evidence reported by Patel et al. (2018) indicates a high prevalence of emotional and behavioral difficulties (40.8%), anxiety symptoms (29.8%) and depressive symptoms (26.9%) among adolescents in sub-Saharan Africa. AGYW are particularly vulnerable due to intersecting social determinants, including gender-based violence, limited access to education and restrictive gender norms that constrain autonomy and agency. AGYW who experience adverse conditions such as poverty, being out of school, or exposure to traumatic events are disproportionately affected and often report higher levels of psychological distress than their male counterparts or in-school peers (Kuringe et al., 2019).

In Burundi, however, data specific to the mental health status of AGYW remain notably sparse, reflecting a broader regional gap in disaggregated, gender-sensitive mental health research. To date, no peer-reviewed studies have systematically examined the prevalence or severity of mental health disorders among Burundian AGYW. One notable exception is a study that assessed mental health among adolescents attending school in Bubanza and Cibitoke provinces, which reported baseline mean scores of 15.62 for posttraumatic stress symptoms and 9.97 for depressive symptoms (Tol et al., 2014). While these findings suggest comparatively lower symptom severity than some studies conducted in South Africa, the absence of gender disaggregated data limits their interpretive value (Tol et al., 2014; Rossouw et al., 2018).

The lack of comprehensive, context-specific evidence underscores an urgent need for targeted mental health research focusing on AGYW in Burundi. Without such data, the design and implementation of effective, gender-responsive mental health interventions remain severely constrained. Expanding empirical research in this area is essential to inform policies and practices that promote psychological well-being and resilience among this vulnerable population.

Adolescents in sub-Saharan Africa face considerable mental health challenges that are shaped by both structural inequalities and socio-cultural dynamics. Aboagye et al. (2022) emphasized the role of psychosocial factors such as loneliness and low perceived social support, identifying these as significant predictors of suicidal ideation among in-school adolescents. Drawing on data from over 19,000 students across eight countries, their study reported a 14.5% prevalence of suicidal ideation, underscoring the urgent need for comprehensive mental health interventions at both school and community levels. Within this broader context, adolescent girls are disproportionately affected due to gender-based vulnerabilities. Gender disparities in mental health are well-documented, with evidence indicating that adolescent girls experience higher rates of depression, anxiety and self-harm compared to their male counterparts (Kapungu and Petroni, 2017). These disparities are reinforced by restrictive gender norms, limited agency and heightened exposure to gender-based violence. The psychological burden is even more acute among conflict-affected and displaced populations. For instance, in a feasibility study of WHO’s Early Adolescent Skills for Emotions (EASE) intervention among Burundian refugee adolescents in Tanzania, Fine et al. (2021) reported elevated levels of depression, anxiety and Post-Traumatic Stress Disorder (PTSD), highlighting the need for contextually relevant and culturally sensitive interventions.

In Burundi, emerging research points to a troubling prevalence of severe mental health conditions among adolescent girls. Charak et al. (2017) found that over 37% of Burundian girls in their study reported experiencing four or more forms of maltreatment – —including physical, emotional and sexual abuse, as well as neglect – exemplifying the cumulative nature of trauma and its association with increased risk of depression and PTSD. These findings reinforce the importance of adopting a trauma-informed approach to mental health care for adolescents. Additionally, Irankunda et al. (2017) identified local idioms of distress, such as *akabonge* (describing symptoms akin to depression) and *guhahamuka* (trauma-related symptoms), which reflect culturally specific understandings of mental health and underscore the need for linguistically and culturally grounded mental health frameworks. Collectively, this body of evidence highlights both the severity of adolescent mental health issues in Burundi and the urgent need for gender-responsive, culturally adapted and evidence-based interventions.

Advancing adolescent mental health in sub-Saharan Africa necessitates the adoption of culturally sensitive and gender-responsive approaches that account for the unique psychosocial realities faced by AGYW. Scalable, evidence-based interventions such as WHO’s EASE program have shown promise, particularly when implemented through task-shifting models that engage non-specialist providers (Hamdani et al., 2024). Despite such innovations, Kapungu and Petroni (2017) caution that many existing mental health programs fail to adequately address the specific needs of AGYW, thereby perpetuating significant gaps in access and quality of care. To address these shortcomings, a holistic and integrated approach is required – one that not only delivers mental health services but also simultaneously promotes educational attainment, gender equity and economic empowerment. Such multidimensional strategies are critical for fostering resilience and improving long-term mental health outcomes among AGYW in resource-constrained and conflict-affected settings.

## Rationale/justification for the study

AGYW in low-resource and conflict-affected settings, such as Burundi, face compounded mental health vulnerabilities shaped by gender-specific stressors, including harmful social norms, early marriage and economic disenfranchisement (Kapungu and Petroni, 2017). Despite increasing global recognition of adolescent mental health as central to achieving AGYW’s overall well-being, interventions in low- and middle-income countries frequently overlook the intersecting social, cultural and structural determinants that uniquely affect AGYW (Singh et al., 2021; Hamdani et al., 2024). Moreover, prevailing mental health programs often lack the contextual and cultural sensitivity necessary for equitable and effective delivery. The experiences of AGYW in Burundi, marked by prolonged conflict, displacement and entrenched gender inequality, highlight the inadequacy of one-size-fits-all approaches and underscore the need for tailored, locally informed strategies.

This study seeks to fill critical knowledge gaps by examining the distinct mental health challenges faced by AGYW in Burundi and exploring the complex interplay of socio-cultural and structural factors shaping their psychological well-being, with particular attention to barriers and facilitators to accessing mental health support. Grounded in the lived experiences of AGYW, the research aims to inform the development of evidence-based, culturally responsive and gender-sensitive mental health interventions. By doing so, it contributes to the growing body of scholarship advocating for more nuanced and inclusive approaches to mental health in LMICs. The study’s findings are intended to provide actionable insights for policymakers, healthcare practitioners and development actors committed to fostering resilience and improving mental health outcomes for AGYW in Burundi and similar contexts.

## Research questions

This study focused on the following research questions to better understand the mental health experiences of AGYW in Burundi:

1. What mental health challenges do AGYW in Burundi experience?
2. What socio-cultural, economic and structural factors shape the mental health and psychological well-being of AGYW in Burundi?
3. What barriers and facilitators influence AGYW’s access to and engagement with mental health support, including formal services, community-based resources and informal support systems?

## Methods

### Study design and participants

This study employed a qualitative research design to investigate the mental health challenges faced by AGYW in Burundi. A total of seven focus group discussions (FGDs) were conducted with 55 AGYW aged 15–24 years. Each FGD comprised seven to eight participants to facilitate in-depth dialog while maintaining a manageable group dynamic conducive to open and meaningful engagement. Participants were recruited through purposive sampling to ensure a diverse representation of AGYW across key variables, including ethnicity and educational background (in-school and out-of-school youth). This sampling strategy was employed to capture the heterogeneity of lived experiences and contextual influences on mental health among AGYW in Burundi.

Participants were eligible if they were AGYW aged 15–24 years residing in the selected study communities and able to provide informed consent. Individuals outside the specified age range or those unable to provide informed consent were excluded from participation. Participants were recruited through community networks established by USAID and its partner organizations. Recruitment was conducted in collaboration with local community organizations, schools and implementing partners, who facilitated access to eligible participants within the study communities. FGDs were conducted with purposively recruited participants until thematic sufficiency was achieved. The number of FGDs was guided by the principle of information power, whereby data collection continued until the discussions yielded sufficient depth and redundancy in themes to adequately address the study objectives, consistent with established qualitative research standards.

The FGDs were guided by a semi-structured interview protocol designed to explore a broad range of mental health-related experiences and challenges. Key areas of inquiry included socio-cultural norms, structural barriers and individual-level factors shaping participants’ psychological well-being. The discussion guide was originally developed in English and subsequently translated into French and Kirundi to ensure cultural and linguistic appropriateness. A back-translation process was used to validate translation accuracy and preserve the meaning of key constructs. The guide included open-ended questions and tailored probes designed to elicit rich, reflective responses. For example, participants were asked about perceived barriers to accessing mental health services (e.g., “What barriers exist for AGYW in Burundi in accessing mental health services?”) and the availability and effectiveness of community or institutional mental health resources (e.g., “What organizations or community resources are you aware of that focus on addressing mental health challenges among AGYW?”). Follow-up prompts examined whether these services were implemented in both educational and community-based settings and encouraged participants to describe the types of programs offered.

### Data collection procedure

Data for this study were collected through FGDs, facilitated by a local Mental Health and Psychosocial Support expert in collaboration with a trained female research assistant with extensive experience working with AGYW in Burundi. This co-facilitation approach was deliberately employed to cultivate a safe, gender-sensitive environment conducive to open dialog, particularly regarding sensitive and gender-specific topics. The pairing of facilitators ensured both cultural competence and methodological rigor, contributing to the trustworthiness of the data collected.

All FGDs were conducted in local languages (Kirundi and French), enabling participants to articulate their experiences and perspectives with clarity and ease. This linguistic accessibility minimized communication barriers and facilitated richer, more authentic data. The discussions were held in community centers and schools selected for their accessibility, safety and privacy, allowing participants to engage comfortably and confidentially. Each session lasted approximately 90 min. Field notes were systematically taken to document non-verbal cues and contextual factors relevant to the discussions.

To uphold the integrity and consistency of the data collection process, the facilitation team engaged in structured debriefing sessions following each FGD. These sessions served to reflect on group dynamics, identify potential facilitator biases and adjust interview techniques as needed, thereby enhancing the reliability and depth of the qualitative inquiry.

### Data analysis

To ensure rigor and credibility in the qualitative analysis, this study employed a structured thematic analysis guided by the six-phase framework proposed by Braun and Clarke (2006) and further elaborated by Kiger and Varpio (2020). This approach provided a systematic process for identifying, analyzing and interpreting patterns within the data. The analysis was conducted using detailed field notes and structured debriefing summaries recorded by trained facilitators who conducted the FGDs. Because the discussions were not audio-recorded and verbatim transcripts were not produced, the field notes served as the primary data source for coding and analysis; this methodological limitation is further described in the limitations section.

Codes were generated inductively from the field notes and debrief summaries to ensure that the analysis remained grounded in participants’ reported experiences. The coding process involved multiple iterative rounds of review and refinement, allowing researchers to engage recursively with the data and ensure that the codes captured the depth and complexity of participants’ narratives. Multiple members of the research team independently coded the field notes to enhance analytic rigor. Following the initial coding process, coders compared their coded materials through iterative review to assess consistency in code application. Rather than calculating a formal statistical measure of inter-coder reliability, agreement was assessed through systematic comparison and consensus-based discussions, consistent with common practices in qualitative thematic analysis. Any discrepancies in code interpretation or application were resolved through deliberation among the coders, and the coding framework was refined as needed to ensure consistent and coherent application of codes across the dataset.

Themes were subsequently developed inductively, allowing patterns and meanings to emerge directly from the data rather than being constrained by predetermined theoretical frameworks. Both semantic (explicit) and latent (underlying) meanings were examined to capture the social, emotional and structural dimensions influencing the mental health experiences of AGYW. The analysis was conducted within a constructivist paradigm, which views knowledge as co-constructed through the interaction between researchers and participants. This epistemological orientation informed both the analytic process and the interpretation of findings, enabling a contextualized understanding of AGYW’s mental health within the broader socio-cultural landscape of gender dynamics, structural inequality and postconflict recovery in Burundi.

### Ethical considerations

Ethical approval for this study was obtained from the Institutional Review Board (IRB) at the University of North Carolina at Chapel Hill and a local ethics review board in Burundi, ensuring adherence to both international and national standards for research involving human subjects. This dual ethical oversight was critical for upholding the cultural, legal and procedural expectations of both academic and local research environments. Prior to data collection, informed consent was obtained from all participants. They received comprehensive information outlining the study’s objectives, methodology, potential risks and benefits, and the voluntary nature of their participation. Measures were taken to ensure participant confidentiality and anonymity, including the secure storage of data and the removal of identifying information during analysis and dissemination. Participants were also explicitly informed of their right to withdraw from the study at any stage without penalty. These protocols were designed to protect participants’ autonomy and dignity while fostering a safe and respectful research environment consistent with ethical best practices.

### Ensuring rigor

To enhance the credibility and trustworthiness of the study’s findings, a range of rigorous methodological strategies were employed. The use of a structured FGD guide ensured consistency in data collection across all sessions, facilitating comparability and coherence. Regular debriefing sessions and reflexive discussions between facilitators were integral to identifying and mitigating potential researcher biases. Additionally, peer debriefing among members of the research team provided critical external perspectives, contributing to a more comprehensive and balanced interpretation of the data. Collectively, these strategies grounded the analysis in the lived experiences of AGYW in Burundi, enabling a nuanced understanding of the mental health challenges they face within their specific socio-cultural and structural contexts.

Thematic analysis, following Braun and Clarke’s (2006) six-phase framework, served as the primary analytical approach. Rather than relying on direct quotations, the analysis focused on the systematic identification, categorization and synthesis of emergent themes across FGDs. This inductive approach enabled a nuanced interpretation of participant narratives while minimizing distortions that could arise from translation and transcription challenges. The data analyzed in this study also represent a subset of a larger research initiative, allowing for triangulation across multiple data sources to enhance the credibility of the thematic findings. By comparing patterns across field notes, facilitator reflections and debriefing summaries, the research team was able to cross-validate themes and identify converging insights, thereby strengthening analytical trustworthiness.

### Limitations

Given that the FGDs were conducted in local languages (Kirundi and French) and subsequently translated into English, the study did not incorporate direct participant quotations in the final analysis. This decision was made to mitigate the risk of misrepresenting participants’ perspectives due to challenges associated with literal translation, including the absence of equivalent terminology in English and linguistic inconsistencies across regional dialects. While the omission of verbatim quotes may be viewed as a limitation, multiple methodological strategies were employed to preserve the rigor, validity and authenticity of the findings.

Ethical considerations further shaped data collection procedures. Due to the highly sensitive nature of the topics discussed, including personal and family-related experiences and the pervasive stigma surrounding mental health in the local context, participants did not consent to audio recording. Respecting these ethical boundaries was critical to fostering an open and safe environment for disclosure. However, this constraint posed methodological challenges, particularly the inability to revisit discussions for verbatim validation.

To address these limitations, a trained local mental health and psychosocial support expert was designated as the primary note-taker. This individual possessed both contextual fluency and subject-matter expertise, allowing for detailed and culturally attuned documentation of participant responses. The note-taking process was guided by a structured FGD protocol to ensure consistency across sessions. Additionally, to minimize recall bias and improve data fidelity, immediate post-FGD debriefings were conducted with facilitators and the note-taker. These debriefings involved the systematic review and clarification of field notes to ensure that key discussion points were accurately captured while still fresh in memory. Reflexivity was maintained throughout the research process, with the team engaging in continuous critical reflection on their positionalities and potential biases. These reflexive practices, coupled with methodological triangulation and structured documentation, contributed to a robust and ethically grounded analysis of the lived experiences of AGYW in Burundi.

## Results

The findings explore emergent themes within three domains: Conditions and overarching factors related to mental health, barriers to improved mental health for AGYW and interventions and facilitators to improve mental health for AGYW. As the FGDs were conducted in local languages (Kirundi and French) and subsequently translated into English, direct participant quotations were not incorporated into the final analysis.

**Conditions and overarching factors related to mental health:** The domain of conditions and overarching factors related to mental health highlights the experience and prevalence of mental health challenges among AGYW in Burundi and underscores a critical gap in recognition of mental health issues, support and intervention. The frequent reporting of anxiety, depression, stress, grief and social isolation among AGYW suggests that poor mental health is a widespread issue. However, FGDs revealed that there is a tendency to perceive these experiences as typical mood fluctuations rather than legitimate mental health concerns, indicating a lack of awareness and mental health literacy. Participants frequently mentioned that this normalization leads to reluctance and delays in seeking support and reinforces patterns of intentional social distancing.

Persistent mood disturbances were described as a serious challenge for girls in school, often reducing their participation, increasing their absences, and, in some cases, leading to dropping out. Participants mentioned that the more severe manifestations of mental illness, such as erratic behavior, disorganized speech and aggression, are met with significant stigma, further exacerbating exclusion from both formal education and essential social support networks. This response reflects broader societal attitudes that frame severe mental health symptoms as threats rather than treatable conditions, reinforcing cycles of marginalization.

### Barriers to improved mental health for AGYW

Within the domain of barriers to improved mental health for AGYW, family dynamics, school environment, cultural beliefs and stigma, and systemic and structural barriers were identified as emergent themes.

#### Family dynamics

Family dynamics play a pivotal role in shaping the mental health trajectories of AGYW in Burundi. Key factors such as parental neglect, intra-family conflict and the absence of emotional support were identified as major contributors to psychological distress. Participants reported that AGYW from divorced or separated households often experience heightened emotional instability and disrupted family structures, which, in turn, may precipitate maladaptive coping strategies such as substance use and engagement in transactional or survival sex. These behaviors reflect both the depth of unmet emotional and psychological needs and the inadequacy of protective support systems within their familial environments.

Particularly concerning were accounts from AGYW residing with stepparents or in foster care arrangements, where the risk of physical, emotional and sexual abuse was markedly elevated. Such experiences not only intensify pre-existing mental health conditions but also perpetuate cycles of trauma and psychosocial vulnerability. Parental rejection emerged as an additional stressor, further eroding AGYW’s sense of self-worth and belonging, and contributing to heightened feelings of loneliness, hopelessness and emotional isolation.

Cultural practices and gender norms further compound these familial stressors. The preferential treatment of male children, a norm deeply embedded in many Burundian households, leads to reduced investment in AGYW’s education and emotional well-being. This systemic gender bias limits AGYW’s access to familial resources, emotional validation and decision-making power, leaving AGYW feeling marginalized and undervalued.

Communication barriers within families, especially between AGYW and male caregivers, further hinder the formation of supportive relationships. The lack of open, empathetic dialog diminishes opportunities for AGYW to express their concerns and access emotional support, thereby limiting the development of healthy coping mechanisms.

#### School environment

The school environment represents a pivotal context for shaping the mental health outcomes of AGYW in Burundi, functioning simultaneously as a setting for academic development and a source of psychosocial stress. FGDs revealed that mistreatment by teachers, manifested through verbal abuse, derogatory comments and perceived unfair assessments, contributed to a hostile educational climate. The pervasive fear of corporal punishment further exacerbated psychological distress, fostering a sense of insecurity and devaluation among AGYW. Such adverse interactions with authority figures were reported to diminish self-esteem, reduce engagement in academic activities and elevate emotional and behavioral difficulties.

Peer-based violence, particularly bullying, emerged as another salient stressor within the school setting. AGYW who lacked access to menstrual hygiene products reported frequent instances of stigmatization and ridicule, which not only undermined their dignity but also discouraged consistent school attendance. This ridicule, compounded with inadequate sanitation infrastructure and the prohibitive cost of menstrual supplies, contributed to a pattern of chronic absenteeism. In more severe cases, this translated into permanent school dropout, restricting future educational and economic opportunities. The cumulative burden of teacher mistreatment, peer bullying and poor menstrual hygiene support had deleterious effects on AGYW’s academic performance, psychological well-being and overall school participation.

#### Cultural beliefs and stigma

Beyond the school setting, broader socio-cultural and structural factors profoundly shape AGYW’s experiences with mental health. Deeply entrenched community stigma continues to frame mental illness in terms of moral failure or deviance, discouraging help-seeking behaviors. In the Kirundi language, for instance, mental health challenges are frequently equated with notions of madness or irrationality, further perpetuating harmful stereotypes. Gendered interpretations of mental illness create divergent consequences: while male adolescents are often viewed as socially dangerous, AGYW are perceived as susceptible to exploitation, particularly sexual abuse. Such perceptions intensify AGYW’s marginalization and limit their access to protection and care.

Spiritual attributions of mental illness, such as beliefs in demonic possession or curses, remain pervasive, often leading families to prioritize traditional healing practices over professional mental health services. These tendencies are reinforced by barriers such as the high cost of care and the stigma that some health providers may direct toward individuals of lower socioeconomic status. As a result, AGYW from low-income households are disproportionately excluded from care. By contrast, those from relatively affluent families are more likely to access services and resources.

Cultural taboos surrounding menstruation also have profound implications for AGYW’s mental health and educational continuity. Menstruating girls are frequently excluded from routine activities due to cultural perceptions of impurity. This isolation causes feelings of humiliation, shame and reduced self-esteem. Moreover, the cultural silencing of gender-specific topics, particularly around menstruation and sexual health, reinforces stigma and shame, restricting AGYW’s ability to seek guidance or support during critical developmental transitions.

#### Systemic and structural barriers

Structural and environmental adversities, including recurrent natural disasters and conflict-induced displacement, amplify the psychosocial burdens faced by AGYW. Participants residing in refugee camps, such as Bwagiriza, described exposure to gender-based violence, including sexual exploitation and physical abuse. Such experiences frequently result in long-term mental health conditions, including depression, anxiety and PTSD. These outcomes are exacerbated by a lack of secure environments and limited availability of psychosocial support services for survivors.

Participants consistently identified the scarcity of mental health services and limited awareness of available resources as major barriers to care. Many schools lack trained mental health personnel and educators often have minimal preparation in recognizing or responding to psychological distress. While some mental health services are provided through non-governmental organizations (NGOs) such as World Vision and Jesuit Refugee Service, their reach remains limited and inconsistent across communities. A significant proportion of AGYW remain unaware of the existence of these services.

### Interventions and facilitators to improve mental health for AGYW

Several initiatives were identified as instrumental in supporting the mental health of AGYW in Burundi, with three primary facilitators emerging from the data. These facilitators were classified as formal interventions and informal support systems.

#### Formal interventions

One of the most impactful is the “School Aunties and Uncles” model, a community-based mentoring initiative developed and implemented by international non-governmental organizations (INGOs) such as World Vision in collaboration with the Burundian Ministry of National Education. This model designates select teachers referred to as “aunts” and “uncles” to serve as trusted mentors, providing psychosocial support to AGYW as they navigate academic and personal challenges. By fostering strong, trust-based relationships between students and educators, this initiative has contributed to the creation of safe and emotionally supportive environments within schools. A key strength of the program lies in the accessibility of mentors, who are available not only during school hours but also in community settings, including their homes. This sustained proximity ensures that support is both consistent and contextually grounded, reducing anxiety and enhancing both mental well-being and academic performance among AGYW.

Furthermore, the program’s designated spaces for emotional processing and reflection have been described as critical for helping AGYW develop healthy coping mechanisms. While teachers are often the first point of contact for AGYW seeking support, particularly female teachers, who are perceived as more approachable and empathetic, their capacity to address complex psychosocial issues is constrained. Teachers often lack the resources to provide financial assistance or to mediate family conflicts, and in some instances, their involvement has led to parental resistance, including the removal or transfer of students. When teachers are unavailable or unable to assist, AGYW turn to the designated “aunts” and “uncles,” whose nonjudgmental and supportive approach has earned them the trust of many students. These mentors not only offer emotional guidance but also provide tangible support, such as facilitating access to school materials and food, and reaching out to students at risk of dropping out. Despite its demonstrated benefits, the implementation of the “Aunts and Uncles” model remains limited, primarily due to funding constraints and the lack of financial incentives for participating educators.

Another key facilitator identified was the provision of menstrual hygiene kits, or “dignity kits,” in schools. Distributed through a partnership between the Ministry of Education and local implementing partners, these kits typically include sanitary pads, soap and other hygiene essentials. Participants emphasized that access to menstrual hygiene products not only addressed a critical physical need but also contributed positively to mental well-being and academic continuity. By helping AGYW manage their menstrual cycles with dignity, these kits support broader goals of educational retention and gender equity.

#### Informal support systems

Outside of formal institutional settings, AGYW identified informal support networks, including friends, female family members, spiritual leaders and romantic partners, as additional sources of emotional support. Peer support groups within schools were identified as a promising avenue for improving mental health outcomes. Participants suggested that such groups could serve as spaces for AGYW to share experiences, develop coping strategies and reduce social isolation. These peer-led structures would complement the existing support provided by teachers, who are often the first point of contact for students experiencing emotional distress. While peer relationships were considered important, disclosure was often limited by fears of stigma and breaches of confidentiality. Within families, AGYW were more likely to confide in mothers or aunts than in fathers, largely due to cultural taboos surrounding father-daughter communication on sensitive topics. Spiritual leaders, such as priests and pastors, were also viewed as trusted confidants who offered spiritual guidance and emotional reassurance through prayer and conversation. Some AGYW reported seeking support from boyfriends or other male peers, particularly those who demonstrated empathy and concern. While these informal support systems offer valuable emotional reinforcement, they often lack the structure, training and capacity required to address the complex mental health needs of AGYW comprehensively.

## Discussion

The findings of this study illuminate the multifaceted mental health needs of AGYW in Burundi, highlighting a complex interplay of individual, familial, institutional and systemic factors that either hinder or facilitate mental well-being. These results align with a growing body of literature from similar low-resource and postconflict contexts, underscoring both entrenched barriers and emergent opportunities for intervention.

A key theme that emerged from participant narratives was the widespread normalization and stigmatization of mental health challenges among AGYW. Despite frequent reports of symptoms associated with anxiety, depression, stress and grief, these experiences were often dismissed as transient emotional fluctuations rather than recognized as indicators of legitimate mental health conditions. This observation is consistent with Hassan et al. (2015), who highlighted how stigma, local explanatory models and cultural understandings of psychological distress can impede recognition of mental health problems and shape help-seeking in conflict-affected populations. The tendency to label individuals with more visible symptoms as “dangerous” or “mad” further entrenches social exclusion and inhibits access to educational and psychosocial support systems, as similarly documented by Patel et al. (2018). These stigmas often lead to social withdrawal and exacerbate feelings of isolation among AGYW.

These findings point to an urgent need for increased awareness and a more nuanced understanding of mental health among AGYW, their families and the broader community. Addressing these challenges requires targeted interventions that integrate mental health education, destigmatization efforts and accessible support systems within both educational institutions and community settings. Without such measures, AGYW facing mental health challenges risk further isolation, educational disruptions and limited opportunities for social and economic advancement.

Family dynamics within the home environment emerged as another critical determinant of mental health outcomes. Participants frequently described experiences of emotional neglect, intrafamilial conflict and abuse, particularly within stepfamilies. These findings support existing evidence linking adverse childhood experiences (ACEs) to negative mental health trajectories (Charak et al., 2017). Moreover, entrenched gender biases in the household, manifesting through the preferential allocation of resources and support to boys, contribute to feelings of neglect, diminished self-worth and long-term psychological distress among girls, a pattern also identified in the work of Devries et al. (2013). These findings point to an urgent need for culturally grounded, gender-responsive interventions aimed at strengthening family communication, challenging discriminatory gender norms and establishing robust protective frameworks to support AGYW experiencing family-related adversity.

Educational institutions were identified as both sources of psychological distress and potential venues for meaningful intervention. Bullying, teacher mistreatment and a lack of adequate facilities, especially during menstruation, were reported to contribute significantly to absenteeism, lowered self-esteem and disengagement from learning. These dynamics are consistent with evidence from other low-income settings that advocate for gender-sensitive approaches to school environments (Sommer et al., 2021). Nonetheless, the “Aunts and Uncles” mentorship program emerged as a particularly promising intervention. By fostering trust and offering both emotional and material support, the program addresses key gaps in the school-based psychosocial support infrastructure. This finding is consistent with studies such Austin et al. (2020), which emphasize the efficacy of mentorship in enhancing the resilience and mental health of marginalized youth. The program’s positive impact underscores the need for broader implementation and institutional investment.

Additional measures to address school-based determinants of mental health include the implementation of teacher training programs focused on positive disciplinary practices, school-wide anti-bullying initiatives and the provision of accessible menstrual hygiene products and facilities. The availability of menstrual health and hygiene resources has been linked to improved school attendance, reduced stigma and anxiety associated with menstruation and enhanced self-confidence among AGYW (Austrian et al., 2021; Hennegan et al., 2022; Ayieko et al., 2025). Without such interventions, educational institutions risk reinforcing systemic inequities and exacerbating the mental health vulnerabilities of AGYW. These findings reinforce the importance of developing formal, culturally appropriate mental health services and expanding access to trained counselors and school-based psychosocial support personnel to meet the diverse and intersecting needs of AGYW in Burundi. Another salient barrier is the limited availability and awareness of professional mental health services, underscoring the urgent need for expanded outreach, mental health education initiatives tailored to local contexts and culturally sensitive interventions that address the socio-cultural and economic barriers to mental health care. Participants reported that most schools and communities lack trained mental health professionals, with many AGYW unaware of existing resources. These findings reflect broader global trends highlighted by Lund et al. (2010), who note a profound inequity in the distribution of mental health services in low- and middle-income countries. Despite these structural limitations, AGYW frequently turn to accessible and trusted adults, particularly teachers and mentors, for support, underscoring the need to scale and formalize such community-based interventions.

Outside formal institutional settings, AGYW also draw upon informal support networks, including peers, family members, spiritual leaders and romantic partners. However, stigma and concerns about confidentiality often inhibit open disclosure, particularly among peers. This is in line with findings by Pretorius et al. (2019), who emphasize the importance of cultivating safe, nonjudgmental spaces in which adolescents can openly discuss mental health challenges. The establishment of peer support groups was identified as a key recommendation.

Overall, these findings underscore the complex and interrelated factors influencing the mental health and educational trajectories of AGYW in Burundi, highlighting the necessity of a holistic, culturally responsive approach to mental health for AGYW in Burundi. Effectively addressing these challenges necessitates a multifaceted, contextually grounded approach that integrates stigma reduction, critical engagement with harmful cultural norms, and the provision of accessible, gender-responsive mental health education and services. Interventions should be tailored to the specific vulnerabilities faced by AGYW, with coordinated efforts across schools, families, communities and health systems to foster environments that promote psychological well-being, educational attainment and long-term resilience. Interventions should prioritize the expansion of school-based programs such as the “Aunts and Uncles” mentorship initiative and the establishment of peer support groups. Efforts to raise mental health literacy, combat stigma and train educators in child protection and supportive communication strategies are equally vital. Moreover, these strategies should be complemented by systemic investments to improve the availability and distribution of trained mental health professionals. The documented success of menstrual hygiene interventions in improving school attendance also illustrates the potential of practical, targeted solutions to address broader psychosocial and educational challenges. Collectively, these interventions offer a pathway toward more equitable and responsive mental health systems that support the well-being and educational success of AGYW in Burundi.

## Conclusion

This study highlights the complex, multi-layered determinants shaping the mental health and educational experiences of AGYW) in Burundi. The findings demonstrate that mental health challenges are not only widespread but are also frequently normalized, stigmatized and insufficiently addressed within familial, educational and community contexts. Intersecting influences, including adverse family dynamics, gendered cultural norms, school-based stressors and limited access to mental health services, collectively reinforce cycles of psychological vulnerability, social exclusion and disrupted educational trajectories.

Addressing these challenges requires a holistic, multi-level and culturally grounded response. School-based platforms represent critical entry points for intervention, particularly through the expansion and institutionalization of initiatives such as the “Aunts and Uncles” mentorship program, the establishment of peer support groups, and the integration of trained psychosocial support personnel within schools. At the same time, strengthening teacher capacity in child protection, gender-sensitive engagement and supportive communication is essential to transforming schools into safe and inclusive environments that promote both mental well-being and academic engagement.

Beyond the school setting, community-level interventions are necessary to address deeply entrenched stigma, harmful cultural beliefs and structural inequities that limit help-seeking and access to care. Efforts to improve mental health literacy, challenge discriminatory gender norms and promote open dialog around mental health and reproductive health issues are critical. These must be complemented by systemic investments to expand the availability, accessibility and equitable distribution of trained mental health professionals, particularly in underserved and rural settings.

Importantly, the findings also underscore the value of practical, context-specific interventions. The demonstrated impact of menstrual hygiene support on school attendance and psychosocial well-being illustrates how targeted, resource-sensitive strategies can address both immediate needs and broader structural barriers. Similarly, strengthening informal support systems, including peer networks, families and trusted community figures, offers an opportunity to extend care beyond formal service delivery structures, provided these actors are supported with appropriate training and guidance.

Ultimately, a coordinated and contextually responsive approach that bridges schools, families, communities and health systems is essential. Such an approach holds significant potential not only to improve mental health outcomes but also to enhance educational attainment, advance gender equity and promote long-term social and economic empowerment for AGYW in Burundi.

## Supporting information

10.1017/gmh.2026.10233.sm001 Chowa et al. supplementary material Chowa et al. supplementary material

## Data Availability

Due to the sensitive nature of qualitative data collected from AGYW participants discussing trauma and mental health experiences, the full transcripts are not publicly available. De-identified excerpts or summaries of the data may be shared upon reasonable request to the corresponding author, subject to ethical approval and participant confidentiality considerations.

## Supplementary material

The supplementary material for this article can be found at http://doi.org/10.1017/gmh.2026.10233.

## References

[r1] Aboagye RG, Ahinkorah BO, Seidu AA, Okyere J, Frimpong JB and Kumar M (2022) In-school adolescents’ loneliness, social support, and suicidal ideation in sub-Saharan Africa: Leveraging Global School Health data to advance mental health focus in the region. PLoS One 17(11), e0275660. doi: 10.1371/journal.pone.0275660. PMID: 36350793; PMCID: PMC 9645589. 36350793 PMC9645589

[r2] Austin LJ, Parnes MF, Jarjoura GR, Keller TE, Herrera C, Tanyu M and Schwartz SEO (2020) Connecting youth: The role of mentoring approach. Journal of Youth and Adolescence 49(12), 2409–2428. 10.1007/s10964-020-01320-z. 32974870

[r3] Austrian K, Kangwana B, Muthengi E and Soler-Hampejsek E (2021) Effects of sanitary pad distribution and reproductive health education on upper primary school attendance and reproductive health knowledge and attitudes in Kenya: A cluster randomized controlled trial. Reproductive Health 18(1), 179. 10.1186/s12978-021-01223-7. 34465344 PMC8406733

[r4] Ayieko P, Torondel B, Renju J, Rubli J, Mcharo O, Luwayi JR, Thomas KA, Greco G, Kapiga S and Okello ES (2025) A multifaceted menstrual health intervention to improve psychosocial outcomes and menstrual practices among secondary schoolgirls in Northwest Tanzania: A pilot intervention study. BMC Women’s Health 25(1), 190. 10.1186/s12905-025-03723-1. 40251620 PMC12007168

[r5] Braun V and Clarke V (2006) Using thematic analysis in psychology. Qualitative Research in Psychology 3(2), 77–101. 10.1191/1478088706qp063oa.

[r6] Charak R, de Jong J, Berckmoes LH, Ndayisaba H and Reis R (2017) Assessing the factor structure of the childhood trauma questionnaire, and cumulative effect of abuse and neglect on mental health among adolescents in conflict-affected Burundi. Child Abuse & Neglect 72, 383–392. 10.1016/j.chiabu.2017.09.009. 28917188

[r7] Devries KM, Mak JYT, Bacchus LJ, Child JC, Falder G, Petzold M, Astbury J and Watts CH (2013) Intimate partner violence and incident depressive symptoms and suicide attempts: A systematic review of longitudinal studies. PLoS Medicine 10(5), e1001439. 10.1371/journal.pmed.1001439. 23671407 PMC3646718

[r8] Familiar I, Sharma S, Ndayisaba H, Munyentwari N, Sibomana S and Bass JK (2013) Community perceptions of mental distress in a post-conflict setting: A qualitative study in Burundi. Global Public Health 8(8), 943–957. 10.1080/17441692.2013.819587. 23941217

[r9] Fine SL, Malik A, Guimond M, Nemiro A, Temu G, Likindikoki S, Annan J and Tol WA (2021) Improving mental health in low-resource settings: A feasibility randomized controlled trial of a transdiagnostic psychological intervention among Burundian refugee adolescents and their caregivers. Behaviour Research and Therapy 145, 103944. 10.1016/j.brat.2021.103944. 34392115

[r10] Hall BJ, Tol WA, Jordans MJD, Bass J and de Jong JTVM (2014) Understanding resilience in armed conflict: Social resources and mental health of children in Burundi. Social Science & Medicine 114, 121–128. 10.1016/j.socscimed.2014.05.042. 24922609 PMC4082029

[r11] Hamdani SU, Huma Z, Malik A, Tamizuddin-Nizami A, Javed H, Minhas FA, Jordans MJD, Sijbrandij M, Suleman N, Baneen U, Bryant RA, Van Ommeren M, Rahman A and Wang D (2024) Effectiveness of a group psychological intervention to reduce psychosocial distress in adolescents in Pakistan: A single-blind, cluster randomised controlled trial. The Lancet Child & Adolescent Health 8(8), 559–570. 10.1016/s2352-4642(24)00101-9. 39025557 PMC11254783

[r12] Hassan G, Kirmayer LJ, Mekki-Berrada A, Quosh C, el Chammay R, Deville-Stoetzel JB, Youssef A, Jefee-Bahloul H, Barkeel-Oteo A, Coutts A, Song S, Alonson C and Ventevogel P (2015) *Culture, Context and the Mental Health and Psychosocial Wellbeing of Syrians: A Review for Mental Health and Psychosocial Support Staff Working with Syrians Affected by Armed Conflict.* Geneva: UNHCR. Available at https://www.escap.eu/uploads/Refugee/13-mental-health-syria-unhcr.pdf (accessed 1 March 2025).

[r13] Hassan G, Ventevogel P, Jefee-Bahloul H, Barkil-Oteo A and Kirmayer LJ (2016) Mental health and psychosocial wellbeing of Syrians affected by armed conflict. Epidemiology and Psychiatric Sciences 25(2), 129–141. 10.1017/s2045796016000044. 26829998 PMC6998596

[r14] Hennegan J, Swe ZY, Than KK, Smith C, Sol L, Alberda H, Bukenya JN, Kibira SPS, Makumbi FE, Schwab KJ and Azzopardi PS (2022) Monitoring menstrual health knowledge: Awareness of menstruation at menarche as an indicator. Frontiers in Global Women’s Health 3, 832549. 10.3389/fgwh.2022.832549. PMC8988033 35400130

[r15] Irankunda P, Heatherington L and Fitts J (2017) Local terms and understandings of mental health problems in Burundi. Transcultural Psychiatry 54(1), 66–85. 10.1177/1363461516689004. 28121243

[r16] Kapungu C and Petroni S (2017) Understanding and Tackling the Gendered Drivers of Poor Adolescent Mental Health. Washington, DC: International Center for Research on Women. Available at https://www.icrw.org/wp-content/uploads/2017/09/ICRW_Unicef_MentalHealth_WhitePaper_FINAL.pdf (accessed March 2025).

[r17] Kieling C, Baker-Henningham H, Belfer M, Conti G, Ertem I, Omigbodun O, Rohde L, Srinath S, Ulker N and Rahman A (2011) Child and adolescent mental health worldwide: Evidence for action. The Lancet 378(9801), 1515–1525. 10.1016/s0140-6736(11)60827-1. 22008427

[r18] Kiger ME and Varpio L (2020) Thematic analysis of qualitative data: AMEE Guide No. 131. Medical Teacher 42(8), 846–854. 10.1080/0142159x.2020.1755030. 32356468

[r19] Kuringe E, Materu J, Nyato D, Majani E, Ngeni F, Shao A, Mjungu D, Mtenga B, Nnko S, Kipingili T, Mongi A, Nyanda P, Changalucha J and Wambura M (2019) Prevalence and correlates of depression and anxiety symptoms among out-of-school adolescent girls and young women in Tanzania: A cross-sectional study. PLoS One 14(8), e0221053. 10.1371/journal.pone.0221053. 31419238 PMC6697336

[r20] Lund C, Breen A, Flisher AJ, Kakuma R, Corrigall J, Joska J, Swartz L and Patel V (2010) Poverty and common mental disorders in low and middle income countries: A systematic review. Social Science & Medicine 71(3), 517–528. 10.1016/j.socscimed.2010.04.027. 20621748 PMC4991761

[r21] Patel V, Saxena S, Lund C, Thornicroft G, Baingana F, Bolton P, Chisholm D, Collins PY, Cooper JL, Eaton J, Herrman H, Herzallah MM, Huang Y, Jordans MJD, Kleinman A, Medina-Mora ME, Morgan E, Niaz U, Omigbodun O, Prince M, Rahman A, Saraceno B, Sarkar BK, De Silva M, Singh I, Stein DJ, Sunkel C, UnÜtzer J (2018) The Lancet Commission on global mental health and sustainable development. Lancet. 392(10157), 1553–1598. doi: 10.1016/S0140-6736(18)31612-X. Epub 2018 Oct 9. Erratum in: Lancet. 2018 Oct 27;392(10157):1518. PMID: 30314863. 30314863

[r22] Pretorius C, Chambers D and Coyle D (2019) Young people’s online help-seeking and mental health difficulties: Systematic narrative review. Journal of Medical Internet Research 21(11), e13873. 10.2196/13873. 31742562 PMC6891826

[r23] Rossouw J, Yadin E, Alexander D and Seedat S (2018) Prolonged exposure therapy and supportive counselling for post-traumatic stress disorder in adolescents: Task-shifting randomised controlled trial. The British Journal of Psychiatry 213(4), 587–594. 10.1192/bjp.2018.130. 29991358

[r24] Shah M, Baird S, Seager J, Avuwadah B, Hamory J, Sabarwal S and Vyas A (2024) Improving mental health of adolescent girls in low- and middle-income countries. Journal of Human Resources 59(S), S317–S364. 10.3368/jhr.1222-12707R2.

[r25] Singh A, Nemiro A, Malik A, Guimond M, Nduwimana E, Likindikoki S, Annan J and Tol WA (2021) Cultural adaptation of a scalable psychological intervention for Burundian refugee adolescents in Tanzania: A qualitative study. Conflict and Health 15(1), 73. 10.1186/s13031-021-00391-4. 34579750 PMC8477522

[r26] Sommer M, Caruso BA, Torondel B, Warren EC, Yamakoshi B, Haver J, Long J, Mahon T, Nalinponguit E, Okwaro N and Phillips-Howard PA (2021) Menstrual hygiene management in schools: Midway progress update on the “MHM in Ten” 2014–2024 global agenda. Health Research Policy and Systems 19(1), 1. 10.1186/s12961-020-00669-8. 33388085 PMC7776301

[r27] Tol WA, Komproe IH, Jordans MJD, Ndayisaba A, Ntamutumba P, Sipsma H, Smallegange ES, Macy RD and de Jong JTVM (2014) School-based mental health intervention for children in war-affected Burundi: A cluster randomized trial. BMC Medicine 12(1), 56. 10.1186/1741-7015-12-56. 24690470 PMC3994237

[r28] Twenge JM and Campbell WK (2018) Associations between screen time and lower psychological well-being among children and adolescents: Evidence from a population-based study. Preventive Medicine Reports 12, 271–283. 10.1016/j.pmedr.2018.10.003. 30406005 PMC6214874

[r29] United Nations Children’s Fund (2021) UNICEF Gender Action Plan, 2022–2025 (E/ICEF/2021/31). United Nations Economic and Social Council. https://undocs.org/E/ICEF/2021/31.

[r30] United States Agency for International Development (2024, May) *USAID Mental Health Position Paper.* Available at https://www.usaid.gov/sites/default/files/2024-05/USAID-MH-Position-Paper_508-compliant_final-May-2024.pdf (accessed September 2024).

[r31] World Health Organization (2004) Promoting mental health: Concepts, emerging evidence, practice (Summary report). World Health Organization. https://iris.who.int/handle/10665/42940 (accessed September 2024).

[r32] World Health Organization (2021, March 30) Mental health: Strengthening our response [Fact sheet]. World Health Organization. Retrieved June 6, 2026, from https://www.who.int/news-room/fact-sheets/detail/mental-health-strengthening-our-response

