# Supplementary material for: Understanding the contextual determinants, barriers and facilitators of mental health for adolescent girls and young women in Burundi: Insights from lived experiences
Source: Glob Ment Health (Camb). 2026 May 28;13:e128. doi: 10.1017/gmh.2026.10233 (PMC13373276; doi:10.1017/gmh.2026.10233)
Supplement: Chowa et al. supplementary material [file S2054425126102337sup001.docx]

## **Appendix 1:** Focus Group Discussion Protocols

**Task 18: USAID/Burundi Education and Mental Health Integration Analysis**

**Focus Group Discussion Protocol: Young women (18+) and/or Parents and caregivers.**

**Facilitator:**

**Date:**

**Discussion Questions**

1. How is MHPSS understood or perceived in Burundi by [name of population, e.g., general population, youth, etc.]
2. How does the community view people with emotional or mental health concerns?
   1. Are AGYW with the same emotional or mental health concerns viewed by the community in the same way?
   2. If different, ask respondents to explain why the differences in perception?
   3. How do you think the community's perception toward AGYW with emotional or mental health concerns affect access to MH learning opportunities and MH services among AGYW?)
3. What are the mental health challenges faced by different segments of AGYW in Burundi?
   1. common mental health challenges among AGYW living with HIV, refugees, returnees, gender-based violence (GBV) survivors, girls who have experienced sexual abuse or exploitation, pregnant mothers, and those who have experience female genital mutilation (FGM), as well as other key populations, such as survivors of torture, trafficking, or exploitation and those who have experienced physical or psychological trauma in Burundi.
   2. What barriers exist for adolescent girls and young women in Burundi in accessing mental health services?
   3. How easy or difficult is it for adolescent girls and young women to find support with emotional or mental health concerns? (Ask if the same level of difficulty/easiness is evident across different segments of AGYW, including those in schools/attending learning programs. If there are differences, ask to explain what are possible reasons for the differences ?
   4. What else could be done to help AGYW with emotional or mental health concerns?
4. What conditions at home contribute to positive MH among AGYW?
   1. If appropriate, ask for their experiences as examples.
   2. What are common stressors at home that may affect MH among AGYW?
5. What common mental health challenges affect educational success among AGYW in Burundi?
   1. How do these mental health challenges act as barriers to educational success among AGYW in Burundi?
6. Who can AGYW with emotional or mental health concerns talk to for help?
   1. Are these individuals at school or community-based organizations where learning activities are held?
   2. Where do AGYW get information about who to talk to for help with their emotional or mental health concerns?
   3. Ask if they know someone (a friend, a classmate, or a family member or relative) who has talked to these individuals.
   4. Also ask respondents to describe the experience of that someone.
   5. how easy or difficult was it to find those individuals?
7. What organizations or community resources are you aware of that focus on addressing mental health challenges among AGYW
   1. Are they implemented both in educational and in community-based settings?
   2. Can you describe these organizations/community resources?
   3. What services or programs do they provide to address MH challenges among AGYW?)
