# Supplementary material for: Understanding the contextual determinants, barriers and facilitators of mental health for adolescent girls and young women in Burundi: Insights from lived experiences
Source: Glob Ment Health (Camb). 2026 May 28;13:e128. doi: 10.1017/gmh.2026.10233 (PMC13373276; doi:10.1017/gmh.2026.10233)
Supplement: Peer review history

# Reviewer report: Review: Understanding the contextual determinants, barriers and facilitators of mental health for adolescent girls and young women in Burundi: Insights from lived experiences — R0/PR2

**DOI:** 10.1017/gmh.2026.10233.pr2

This work makes a valuable contribution to understanding mental health among young women in Burundi. The literature offers a detailed and well-informed context for the issue, with relevant and current data. The qualitative approach provides a deep insight into these young women’s experiences and highlights the key areas that need to be addressed as priorities. The findings have significant implications for psychoeducation and health promotion.

General recommendations:

1. The title should not only consider barriers, but also the facilitators and contextual conditions, to better reflect the content and findings of the study.

2. I suggest maintaining consistency in the study’s purpose statement. The abstract states: “This study explored AGYW’s lived experiences to identify barriers and facilitators to mental health support”, but the rationale/justification for the study states: “This study seeks to fill critical knowledge gaps by examining the distinct mental health challenges faced by AGYW in Burundi and exploring the complex interplay of socio-cultural and structural factors that shape their psychological well-being”.

3. The research questions do not include exploring facilitators to mental health support; there is only one question (question three) that specifically addresses support through mental health interventions. As the question is phrased, it is understood that only mental health formal support (such as interventions) was explored.

Specific recommendations:

1. Support the following sentence with a citation: “Among those most affected are AGYM, who face disproportionate mental health risk due to their exposure to traumatic experiences such as witnessing violence, forced displacement, and the loss of family members during the civil unrest”, p. 1.

2. Define the acronym “USAID” (p. 3).

3. The first and second paragraphs from the section “Prevalence of Mental Health Challenges for AGYW in Burundi” can be integrated into one paragraph.

4. The authors mention “Regional studies indicate…” (p. 3), but only cite one study (Patel et al., 2018).

5. Support the following sentence with a citation: “One notable exception is a study that assessed mental health among adolescents attending school in Bubanza and Cibitoke provinces, which reported mean scores of 15.96 for posttraumatic stress symptoms and 10.62 for depressive symptoms” (p. 4).

6. There is an inconsistency between the ages of the participants mentioned in the abstract (10-19) and the ages mentioned in the methods section (18-24).

7. Important information is missing in the participant’s section (p. 6):

a. Participant characteristics:

i. It is mentioned that purposive sampling was carried out to ensure diverse representation of participants (ethnicity, disability status, educational background); however, data for these demographic characteristics are not presented. The age range is also mentioned, but not the average age of the participants.

b. Recruitment:

i. There is no mention of inclusion and exclusion criteria, no mention of how participants were recruited (how they were accessed), and no mention of how the sample size was established (were criteria such as saturation or information power used?).

8. It should be mentioned in the analysis section that the data were coded directly from the field notes taken by the trained mental health expert who conducted the focus groups, given that the limitations mentioned that there was no recording or transcription.

9. The analysis mentions that an inter-coder reliability was conducted (p. 7). However, the process for establishing the levels of agreement is not described (was a statistical measure such as % or Cohen’s Kappa used? How were the discrepancies resolved?).

10. The abstract states that four thematic domains emerged, using inductive logic. However, these thematic units correspond to the research questions (RQ):

a. Thematic domain 1: Contextual conditions shaping mental health (RQ 2)

b. Thematic domain 2: Barriers to service access (RQ1)

c. Thematic domain 3: Facilitator for improving access

d. Thematic domain 4: Intervention needs (RQ3)

It would be expected that the themes would develop around the domains explored according to the research questions. For example, in the domain of “barriers to service access” (RQ1), some themes might be “family dynamics,” “school environment,” etc. These are the themes that emerge from the data, based on what I could interpret from the results presented. Also, a visual (such as a figure) could help present the themes identified for each domain explored.

11. In the results section, I would separate formal facilitators from informal ones (this would be a separate heading). According to the domain explored, which was “Intervention needs,” corresponding to research question 3, only those related to facilitators or supports in formal settings would be presented. On the other hand, although not included in the research questions, some themes were developed from other types of facilitators (informal support), such as support networks (e.g., friends, spiritual leaders).

12. Section “Data availability statement” (p. 17) mentions that due to the sensitive nature of the qualitative data regarding trauma and mental health experiences, the full transcriptions are not publicly available. However, the limitations mentioned that there was no recording (therefore, no transcripts). I suggest reviewing this statement to avoid confusion.

# Reviewer report: Review: Understanding the contextual determinants, barriers and facilitators of mental health for adolescent girls and young women in Burundi: Insights from lived experiences — R0/PR3

**DOI:** 10.1017/gmh.2026.10233.pr3

**Authors:** Elsa Denise Perestrelo

This manuscript dives into an important and underaddressed global mental health concern. Exploring the mental health experiences of AGYW is highly relevant given the intersection of poverty, gender inequality, conflict, and limited-service infrastructure in the Burundian context. The inductive and qualitative approach provides unique context into the problem and potential solutions to support the unique needs of AGYW in Burundi.

However, the article would benefit from further revision to strengthen its structure, methodological rigor and clarity of research aim and findings.

Abstract:

• While the problem is clearly articulated, the overarching aim of the study remains unclear. Specifically, it is not evident whether the primary objective is (a) to identify barriers and facilitators to mental health support for AGYW (in the abstract), or (b) to examine the mental health challenges faced by AGYW more broadly, as suggested in the rationale (p. 5).

• There is a noticeable discrepancy in reported participant ages. The abstract lists ages 10–19 years old, while the Methods section lists participants as ages 18–24. This requires clarification, as it affects the framing of AGYW and interpretation of findings.

Background:

• The authors should clearly define the age range encompassed by the term AGYW, as it is a key aspect of the study. This is important, given the developmental and contextual differences between adolescents and young women (see, for example: https://doi.org/10.1007/s10461-020-02974-3).

• Several claims would benefit from additional citations to strengthen the evidence base (e.g., page 2, lines 33–38, where broad statements are made about mental health burden and service access).

• When referencing prior studies, it would be helpful to specify the geographical context of the research (e.g. East Africa or other sub-Saharan African regions). This would improve the relevance of cited findings.

• The authors may wish to consider relocating the paragraphs on page 4 (lines 12–30) to the rationale section, as they more directly justify the need for the current study rather than providing background context.

• It remains unclear whether the manuscript focuses primarily on adolescent girls, young women, or both. For instance, page 4 emphasizes adolescent mental health challenges, while comparable discussion specific to young women is limited. Please clarify the intended population and ensure balanced coverage aligned with the stated AGYW focus.

Methods:

• Additional detail on the study setting would strengthen contextual understanding. Please specify where in Burundi the study was conducted and whether the setting was primarily rural, urban, or mixed.

• Kindly consider adding the semi-structured interview protocol as a supplementary material. Additionally, please clarify whether the referenced structured FGD guide is standardized or study-specific.

• More detail is needed regarding data management and analysis. For example, how was data stored, and was coding conducted manually or using qualitative analysis software?

Results:

• The Results section would benefit from a brief description of participant characteristics (e.g., mean age, school enrollment status, in-school vs. out-of-school participants).

• Authors should ensure that the Results section is limited to reporting findings only. Interpretive statements should be reserved for the Discussion (e.g., page 11, lines 42–50).

• Consider adding paraphrased translations of some of the participants´answers in the findings and or direct quotes in Kirundi and French with translations in brackets.

• Consider restructuring how results are presented to avoid repetitions of findings. For instance, the repeated mention on implications of cultural views on menstruation as a mental health barrier.

Discussion:

This section clearly dissects the manuscript´s findings and is well written. To further strengthen the section, authors should consider adding a paragraph on study limitations such as researcher bias associated with purposive sampling.

Authors should consider the moving some discussion paragraphs from the results to discussion section.

Conclusion:

The conclusion is concise and well written.

Consider adding one paragraph that summarizes the manuscript’s findings in relation to its central aim and thematic areas highlighted in the findings.

# Editor report: Recommendation: Understanding the contextual determinants, barriers and facilitators of mental health for adolescent girls and young women in Burundi: Insights from lived experiences — R0/PR4

**DOI:** 10.1017/gmh.2026.10233.pr4

**Affiliations:** Ponce Health Sciences University, Puerto Rico

No accompanying comment.

# Editor report: Decision: Understanding the contextual determinants, barriers and facilitators of mental health for adolescent girls and young women in Burundi: Insights from lived experiences — R0/PR5

**DOI:** 10.1017/gmh.2026.10233.pr5

**Affiliations:** London School of Hygiene & Tropical Medicine, United Kingdom of Great Britain and Northern Ireland

No accompanying comment.

# Reviewer report: Review: Understanding the contextual determinants, barriers and facilitators of mental health for adolescent girls and young women in Burundi: Insights from lived experiences — R1/PR7

**DOI:** 10.1017/gmh.2026.10233.pr7

**Authors:** Elsa Denise Perestrelo

The authors have done a wonderful job revising this paper. The corrections and additions strengthen the quality of the paper considerably, and the writing and structure is now clear throughout. I am happy to recommend acceptance.

# Editor report: Recommendation: Understanding the contextual determinants, barriers and facilitators of mental health for adolescent girls and young women in Burundi: Insights from lived experiences — R1/PR8

**DOI:** 10.1017/gmh.2026.10233.pr8

**Affiliations:** Ponce Health Sciences University, Puerto Rico

The rationale for excluding individual level participant data from the manuscript is well addressed in the response letter. However, readers would greatly benefit from reading the rationale as well. I suggest the team to integrate it into the manuscript.

# Editor report: Decision: Understanding the contextual determinants, barriers and facilitators of mental health for adolescent girls and young women in Burundi: Insights from lived experiences — R1/PR9

**DOI:** 10.1017/gmh.2026.10233.pr9

**Affiliations:** London School of Hygiene & Tropical Medicine, United Kingdom of Great Britain and Northern Ireland

No accompanying comment.
